# Glioma cells on the run – the migratory transcriptome of 10 human glioma cell lines

**DOI:** 10.1186/1471-2164-9-54

**Published:** 2008-01-29

**Authors:** Tim Demuth, Jessica L Rennert, Dominique B Hoelzinger, Linsey B Reavie, Mitsutoshi Nakada, Christian Beaudry, Satoko Nakada, Eric M Anderson, Amanda N Henrichs, Wendy S McDonough, David Holz, Anna Joy, Richard Lin, Kuang H Pan, Chih J Lih, Stan N Cohen, Michael E Berens

**Affiliations:** 1 Translational Genomics Research Institute, Phoenix, AZ 85004, USA; 2 Department of Neurosurgery, Kanazawa University, Ishikawa 920-0934, Japan; 3 Department of Human Pathology, Kanazawa University Graduate School of Medicine, Kanazawa, Ishikawa 920-0934, Japan; 4 Department of Genetics, Stanford University School of Medicine, Stanford, CA 94305, USA

## Abstract

**Background:**

Glioblastoma multiforme (GBM) is the most common primary intracranial tumor and despite recent advances in treatment regimens, prognosis for affected patients remains poor. Active cell migration and invasion of GBM cells ultimately lead to ubiquitous tumor recurrence and patient death.

To further understand the genetic mechanisms underlying the ability of glioma cells to migrate, we compared the matched transcriptional profiles of migratory and stationary populations of human glioma cells. Using a monolayer radial migration assay, motile and stationary cell populations from seven human long term glioma cell lines and three primary GBM cultures were isolated and prepared for expression analysis.

**Results:**

Gene expression signatures of stationary and migratory populations across all cell lines were identified using a pattern recognition approach that integrates *a priori *knowledge with expression data. Principal component analysis (PCA) revealed two discriminating patterns between migrating and stationary glioma cells: i) global down-regulation and ii) global up-regulation profiles that were used in a proband-based rule function implemented in GABRIEL to find subsets of genes having similar expression patterns. Genes with up-regulation pattern in migrating glioma cells were found to be overexpressed in 75% of human GBM biopsy specimens compared to normal brain. A 22 gene signature capable of classifying glioma cultures based on their migration rate was developed. Fidelity of this discovery algorithm was assessed by validation of the invasion candidate gene, connective tissue growth factor (CTGF). siRNA mediated knockdown yielded reduced *in vitro *migration and *ex vivo *invasion; immunohistochemistry on glioma invasion tissue microarray confirmed up-regulation of CTGF in invasive glioma cells.

**Conclusion:**

Gene expression profiling of migratory glioma cells induced to disperse *in vitro *affords discovery of genomic signatures; selected candidates were validated clinically at the transcriptional and translational levels as well as through functional assays thereby underscoring the fidelity of the discovery algorithm.

## Background

Glioblastoma multiforme (GBM) is the most common primary brain tumor, affecting 20,000 patients per year; the peak age of occurrence is between 50–60 years of age. Despite advances in diagnosis and treatment, life expectancy for patients suffering from this disease still remains at 18 months [1]. Both genetic heterogeneity and highly invasive behavior are believed to be responsible for recurrent tumor growth, which occurs typically within 3 cm of the initial resection cavity; these behaviors also contribute to poor therapeutic response [1]. Although invasive cells are recognized as drivers of poor outcome, as they are left behind after surgical debulking [2], no specific treatment has been developed targeting this important tumor cell subpopulation [3-5]. We have recently reported that invasive GBM cells comprise a unique population that is characterized by heightened resistance to induction of apoptosis [6]. While global expression profiles of glial tumors have been studied extensively, less is known about gene expression in invasive glioma [7,8]. To shed light on the biological processes that drive invasive behavior and to identify novel candidates that may serve as targets for specific anti-invasive therapies, we sought to develop a discovery approach that can be applied to an *in vitro *model system of glioma migration. Gene expression profiles of a panel of seven established glioma cell lines and three primary glioma cultures, induced to migrate for 24 hours, were established. They revealed two signatures of migrating and stationary glioma cells and selected candidates were validated clinically on a comprehensive glioma expression data set, a glioma invasion tissue microarray (TMA) and functionally in migration assays and *ex vivo *rat brain slice assays.

## Methods

### Glioma cell tissue culture

Ten GBM cell lines were selected for this study: seven established (U87MG, T98G (ATCC), U87ΔEGFR [9] G120, G112MS, SF763 and SF767 [10], and three primary cultures (GH3, 4, and 6) that were kindly provided by R. Goldbrunner, Dept. of Neurosurgery, Munich. Germany. All tissue culture was done using 10% FBS MEM and cells were kept at 37°C, 5% CO2 and 100% humidity. Primary cultures were used at or under passage 5, maintained in 20% FBS MEM and switched to 10% FBS MEM twenty-four hours prior to use in migration assay. All cell cultures were tested for, and found to be free of, *Mycoplasma *sp. infection by DAPI staining regularly.

### Radial Migration Assay

The radial migration assay was performed as described previously [11,12]. To simulate a GBM migratory front (rim) and proliferating core, three thousand glioma cells were seeded as a defined, confluent circular monolayer using cell sedimentation manifolds (CSM, Inc., Phoenix, AZ) on glioma-derived extra-cellular matrix coated 10-well slides. Migration was initiated by removing the manifold 4 hours after seeding and cells then radially dispersed for 24 hours. Migration rates were estimated from photomicrographs of migration assays taken at time point 0 hours and time point 24 hours [13].

For microarray experiments, stationary (core) and migratory (rim), cells were harvested under an inverse microscope (Axiovert 100, Zeiss, NY) using P2 pipette in three independent biological replicates. Thirty individual dispersion assays (three 10-well slides) were collected per cell line and all materials were stored at -20°C until RNA isolation. Core and rim cells were separately lysed with Trizol (Invitrogen, Carlsbad, CA).

### RNA isolation, amplification and labeling

Total RNA was extracted from Trizol solution, according to manufacturer's instructions and followed with purification through RNeasy mini kit (Qiagen, Valencia, Ca). Total RNA quality and quantity were assessed by Bioanalyzer RNA 6000 Nano LabChip Kit (Agilent Technologies, Palo Alto, CA). Samples selected for the study contained intact RNA as determined by visual inspection of electropherograms for distinct 18S and 28S ribosomal peaks. Samples that failed this assessment were not included in the study.

Sample (either rim or core) and human universal reference RNA (Stratagene, La Jolla, CA) underwent one round of linear amplification and fluorescent labeling in Agilent's Low-Input Linear Amplification and Labeling Kits yielding fluorescent cRNA. Sample RNA was labeled with Cyanine 5 (Cy5) while reference RNA was labeled with Cyanine 3(Cy3) – CTP (PerkinElmer, Boston, MA) and cRNA concentration was assessed spectro-photometrically (GeneQuant II, Bottom Ltd., Cambridge, UK). One hundred nanograms of total RNA from each sample were labeled for hybridization to microarray chips and remaining unamplified sample RNA was saved for validation studies. Reference RNA was amplified/labeled in replicate reactions and pooled.

### Microarray processing and quality testing

Each Cy5-labeled core and rim sample (three biological replicates) was hybridized against Cy3-labeled universal reference RNA on whole human genome 40 K oligonucleotide microarray chips (Agilent) according to manufacture's protocol [14]. Agilent DNA microarray scanner and Feature Extraction Software with default settings for oligonucleotide expression arrays were used to capture and process array images. Spot intensities were extracted and preprocessed by subtracting background noise and applying a LOWESS (locally weighted linear regression) correction for dye-bias [14]. Preprocessed data was then filtered by removing genes with a background signal higher than feature signal. Log_2 _(S/R) (S = sample (rim or core) and R = universal reference) for each gene was calculated.

Technical variation (i.e. variation due to instrumentation, assays and reagents) was assessed with two sets of technical triplicates. One collected at the beginning of the study and one at the end. A comparison of variation in technical and biological triplicates from the same cell line (T98G) was done to determine if technical variation was the same or greater than biological variation. Variation was qualitatively assessed by overlaying standard deviation versus average gene expression for each set of technical triplicates with their corresponding biological replicates. Expression values were log_2 _ratio of ratios, which is calculated by dividing rim ratio (rim/universal reference) by core ratio (core/universal reference).

### Data analysis

A minimum fold change threshold was established by calculating the median standard deviation of rim/core expression for each biological replicate (n = 3). Genes that were not statistically (p > 0.1) differentially expressed were identified with ANOVA and removed. Genes that are not differentially expressed are considered non-informative and removed to reduce the complexity of the data.

In order to identify genes whose expression was consistently up- or down-regulated in rim cells as compared to their corresponding core cells across all cell lines, a pattern recognition approach integrating *a priori *knowledge about transcriptional differences between rim and core populations was employed. Principal component analysis (PCA) was used to determine components of variation in the data whereby patterns can be identified that discriminate samples (i.e. rim from core) [15,16]. Genes whose profiles correlate (r > 0.6 and are visually similar) with the discriminatory PCA-derived patterns serve as probands and are used in the proband-based rule function within the Genetic Analysis By Rules Incorporating Expert Logic (GABRIEL), a platform of knowledge-based algorithms that incorporate biological knowledge to enable systematic microarray analysis, to find sub-sets of genes with similar expression patterns [17]. One of GABRIEL's functions can readily identify additional genes whose expression profiles correlate with those of pre-selected proband genes. Both false discovery and false negative rates (FDR and FNR, respectively) are used to estimate significance of selected genes compared to random chance [18]. Proband analysis was performed with the correlation coefficient threshold set at 0.8 for each proband. Genes identified were categorized according to their Gene Ontology into classes deemed significant for glioma biology.

Migration rates from the replicates of the 7 glioma cell lines and 3 primary cultures were averaged and binned into fast (>average migration rate) and slow migration (<average migration). Significance analysis of microarrays (SAM) was carried out on the union of migratory and stationary signatures derived from GABRIEL in order to identify significantly different genes between fast and slow migration groups. Support vector machines (SVM) with a linear kernel using leave-1-out crossvalidation were used to predict migration rate (e.g. fast or slow) based on gene signatures. A permutation test (10,000 iterations) was performed to assess the performance of the classification.

Clustering of the migration rate signature in the malignant astrocytoma cases was performed using k-means and visualized using multidimensional scaling (MDS) plots. Kaplan-Meier analysis were used to assess survival differences between clusters.

TM4 [19] was used for SAM. Survival analysis was done with SPSS (SPSS Inc., Chicago, IL). ANOVA, PCA, SVM, k-means, and MDS was performed with MATLAB (MathWorks, Inc. Natick, MA)

### Gene expression profiling of human brain tumor specimen

Gene expression data of 111 glial tumors and 24 normal brain specimens was kindly provided by Dr. T. Mikkelsen, Henry Ford Hospital, Detroit MI and Dr. H. Fine Neuro Oncology Branch, National Cancer Institute, National Cancer Institute, Bethesda, MD. Array data was processed according to Affymetrix^® ^MAS5 (Microarray Suite 5) algorithm implemented in Affymetrix^® ^GCOS (GeneChip Operating Software). Text files exported from GCOS were uploaded into GeneSpring^®^7.2 for data management (Silicon Genetics, Redwood City, CA).

Gene symbols and accession numbers were used to map Agilent probes to Affymetrix probes using Affymetrix's Netaffx tool.

### QRT-PCR Validation

QRT-PCR was performed with the unamplified RNA collected for use in microarray experiments. One hundred nanograms of RNA from each biological replicate were reverse transcribed (Supersrcipt III, Invitrogen) using oligo-dT primers. cDNA was diluted 1:4 in water and aliquoted until use. Primers were designed to hybridize in the 3'prime region of the genes and to produce amplicons ranging from 100 to 300 base pairs. Relative quantification was performed on the LightCycler Instrument (Roche, Mannheim, Germany) using LightCycler FastStart DNA Master SYBR Green I and normalized to Histone 3A as a housekeeping gene. Specificity of PCR amplicons was confirmed by melting curve analysis [20] and agarose gel electrophoresis.

Crossing points of target gene vs. housekeeping gene Histone 3A were used to calculate relative fold up-/down-regulation in the invasive cells with the following formula: F = 2^(*IH*-*IG*)-(*CH*-*CG*)^, adapted from [21], where F = fold difference, C = core cells, I = invasive rim cells, G = gene of interest, H = housekeeping (Histone 3A).

### Immunofluorescence

*In vitro *protein expression of CTGF was assessed by immunofluorescence staining of migrating and stationary T98G cells in migration format. After induction of migratory phenotype for 24 hours cells were fixed with 4% paraformaldehyde, permeabilized with 0.5% triton X-100, blocked with 3% goat serum in tris-buffered saline (TBS) for 30 minutes, and incubated at room temperature for one hour with anti-CTGF antibody (SC-25440, Santa Cruz Biotechnology, Inc., Santa Cruz, CA) at 1:100. Addition of Cy3 conjugated secondary antibody (Jackson Laboratories, West Grove, PA) for one hour at room temperature (1:2000) was completed and cells were viewed with a LSM 5 Pascal laser scanning confocal microscope (Zeiss, Thornwood, NY).

### Immunohistochemistry

A glioma invasion specific TMA [7] was used for immunohistochemical evaluation of proteins in stationary and invasive glioma cells. Briefly, the TMA was heated for 2 hours at 65°C, deparaffinized in xylene and hydrated in a graded alcohol series. Endogenous peroxidases were quenched with 3% hydrogen peroxide in PBS for 15 minutes, followed by antigen retrieval using reveal solution in the Decloacking Chamber (Biocare Medical, Concord, CA). Non-specific binding was blocked with 10% normal goat serum in 0.1% Triton X-100 TBS for 1 hour at room temperature. Slides were then incubated with CTGF antibody (1:200) at 4°C overnight, washed and secondary antibody (Vectastain Kit; Vector Laboratories, Burlingame, CA) was applied at room temperature for 1 hour. Slides were exposed to diaminobenzidine (Sigma, St. Louis, MO) for 1 minute and counterstained with hematoxylin 2 (Richard-Allen Scientific, Kalamazoo, MI). TMA was evaluated by a pathologist (S.N.) where percentage of cells exhibiting no(0), weak(+), moderate(++) and strong immunopositivity(+++) was assessed as described previously [7,22]; Pearson chi-square test was employed to compare staining intensity in rim and core population.

### Functional studies

Purified, duplexed siRNAs for CTGF and for luciferase (control) were purchased from Qiagen (Valencia, CA). The siRNA sequences targeting human CTGF (GenBank accession number NM_001901) were GTCCCGGAGACAATGACATCT (C1) and ATCGGAATCCTGTCGATTAGACT (C2). The sequences were designed to be unique when compared with the sequence of other CCN members. The sequence targeting luciferase was AACGTACGCGGAATACTTCGATT. Glioma cells (8 × 10^5^) were transfected with 20 nM of siRNA using lipofectamine 2000 (Invitrogen) and cultured for 48 hours prior to use.

*In-vitro *migration assays were performed on 10-well slides coated with 10 μg/ml laminin.

### *Ex Vivo *Invasion Assay on Rat Brain Slices

An *ex vivo *invasion assay on rat brain slices was carried out as described previously [13,23]. Briefly, 400 μm thick sections were prepared from Wistar rat (Crl:(WI)BR; Charles River Lab, Wilmington, MA) cerebrum. Approximately 1 × 10^5 ^glioma cells stably expressing green fluorescence protein (GFP) were gently placed (0.5 μl transfer volume) on the brain slice in 4–6 replicates per condition. After 72 hours, glioma cell invasion into the rat brain slices was quantitated using a LSM 5 Pascal Laser scanning confocal microscope (Zeiss, Thornwood, NY). Serial optical sections were obtained every 5 μm downward from the surface plane to the bottom of the slice. The invasion rate was calculated as described previously [13]. In brief, for each focal plane, the area of fluorescent cells was calculated and plotted as a function of the distance from the surface of the brain slice.

### Antibodies and Immunoblotting

Glioma cells or glioma tissue specimens were lysed in sample buffer as described previously [23] and separated by 10% SDS-polyacrylamide gel electrophoresis, transferred to nitrocellulose membrane (Invitrogen) and probed with specific antibodies. Horseradish peroxidase-conjugated secondary antibodies were detected using a chemiluminescence system (NEN, Boston, MA). Following stripping, membranes were re-probed with α-tubulin antibody. Bound secondary antibodies were detected using a chemiluminescence system (NEN, Boston, MA). CTGF antibody was from Santa Cruz Biotechnology (Santa Cruz, Ca) and α-tubulin antibody was from Upstate Biotechnology (Lake Placid, NY). Horseradish peroxidase coupled secondary antibodies were purchased from Promega (Madison, WI).

## Results

### *In vitro *glioma migration microarray data analysis

**Figure 1:**
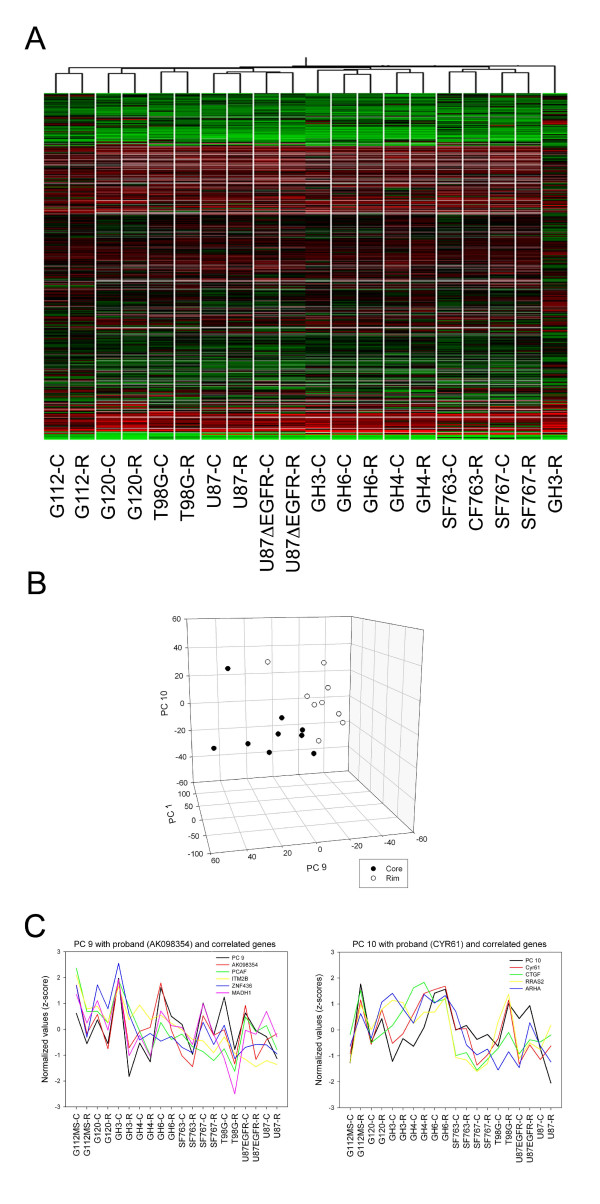
Hierarchical clustering of expression patterns of 20 core and rim samples (**A**). Scatterplot of principal component (PC) 9 against PC10 reveals two clouds representing core (black symbols) and rim samples (white symbols), respectively (**B**). Proband gene AK098354 shows strong correlation with PC9, containing genes down-regulated in rim population (*stationary signature*) while cystein rich 61 (Cyr61) exhibits strong correlation with up-regulation pattern of PC10 (*migratory signature*). GABRIEL was used to detect genes with similar expression patterns to proband genes AK098354 and Cyr61 (**C**).

RNA was isolated from migratory and stationary glioma cells harvested as three biological replicates from the radial migration assay. RNA integrity was confirmed with the Bioanalyzer by the presence of well-defined 18s and 28s ribosomal RNA bands; the median yield per biological replicate was 700 ng. Microarray analysis of migratory glioma cells compared to their stationary cognates was performed using the three biological replicates derived from seven glioma cell lines and three primary cultures. Median standard deviation of rim/core expression for three biological replicates was ± 1.23-fold with a 95% confidence interval at ± 1.5-fold, leading to a detection limit of ± 1.5-fold change between rim and core samples for this study. Hierarchical clustering of median expression for each core or rim sample in this study revealed close similarity between core and rim samples derived from the same cell line (Figure 1A).

An ANOVA-filter removed 25,221 genes that were not statistically differentially expressed across all the samples leaving a subset of 16,454 differentially regulated genes (p <= 0.1) in at least one sample.

PCA was performed on a 16,454 genes × 20 samples matrix. Expert analysis of principal components (PC) revealed that PCs 1–8, comprising the majority of the variation in the data, described the variation between cell lines. PC 9 and 10 exhibited patterns matching anticipated differences in expression between migratory and stationary cells (i.e. up- or down-regulation). PC 9 displayed a down-regulation profile (i.e. rim sample compared to corresponding core) and PC10 displayed an up-regulation profile (rim to core). In addition, plotting the scores of PC 9 and PC 10 against each other formed two clusters, which discriminated rim from core samples (Figure 1B). Proband gene selection identified two genes, AK098354 and CYR61, exhibiting strong correlation of r = 0.71 and 0.64 with PC 9 and 10, respectively (Figure 1C).

The dataset of 16,454 genes was up-loaded into GABRIEL and proband analysis using a correlation coefficient of 0.8 identified a subset of 105 genes correlating with down-regulation pattern of AK098354 (FDR = 0.003 and FNR = 0.889) and will be referred to as "stationary signature"; a subset of 50 genes was found to correlate with up-regulation pattern of CYR61 (FDR = 0.004 and FNR = 0.025) and will be referred to as "migratory signature" [see Additional file 1].

### Technical validation of gene expression data

**Figure 2:**
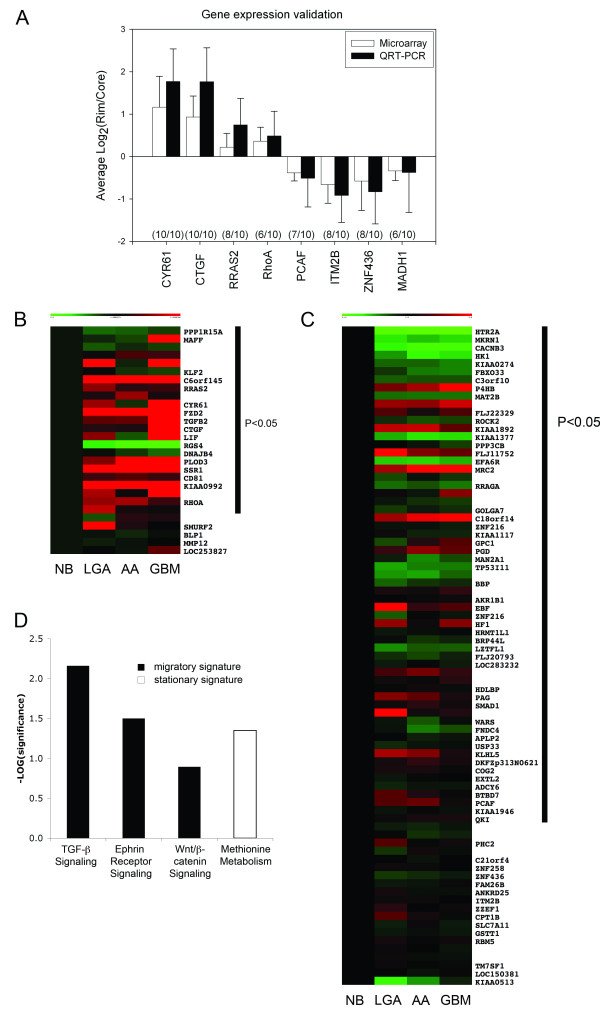
Technical validation of microarray data by quantitative RT-PCR (QRT-PCR). Average Log2 expression of relative mRNA copy numbers derived from three replicate microarray experiments and QRT-PCR of migratory (rim) over stationary (core) glioma cells (error bars = SD). Concordance between directionality of differential regulation between migratory and stationary cells for microarray and QRT-PCR data is displayed in parentheses (CYR61 = cystein rich 61, CTGF = connective tissue growth factor, RRAS2 = related RAS viral oncogene homolog 2, RhoA = ras homolog gene family member A, PCAF = p300/CBP-associated factor, ITM2B = integral membrane protein 2B, ZNF436 = zinc finger protein 436, MADH1 = mothers against decapentaplegic homolog 1 (**A**). Expression pattern of *migratory *(**B**) and *stationary signatures *(**C**) in comprehensive glioma expression data set (NB = normal brain, LGA = low grade astrocytoma, AA = anaplastic astrocytoma, GBM = glioblastoma multiforme). Bar indicates genes significantly (P < 0.05) differentially expressed between tumors and normal brain. Canonical pathways significantly over-represented in *migratory *(black bars) and *stationary signature *(white bar) (**D**).

From each group of *migratory *and *stationary signatures *genes were selected for validation by quantitative RT-PCR because they exhibited 1.5-fold or greater differential expression between rim and core in a majority of the cell lines and are annotated as being engaged in cancer, migration, and/or invasion (literature mining). Cyr61 and CTGF were found to exhibit total concordance of directionality of expression between microarray and QRT-PCR expression. RRAS and RhoA were found to be up-regulated in eight and six, respectively, of the cell lines examined. Integral membrane protein 2B (ITM2B) and zinc finger protein 436 (ZNF436) showed concordant down-regulation between microarray data and QRT-PCR in 8 out of 10 cell lines, while p300/CBP-associated factor (PCAF) and mothers against decapentaplegic homolog 1 (MADH1) showed down-regulation in seven and six out of 10 cell lines, respectively (Figure 2A).

### Clinical validation of migration signatures

*Migratory *and *stationary signatures *were analyzed in a comprehensive expression dataset of 111 glial tumors (8 LGA, 22 AA, and 81 GBM) and 24 non-tumor tissues. 75% of genes in the *migratory signature *(Figure 2B) and 73% in the *stationary signature *(Figure 2C) were found to be significantly (p < 0.05) differentially regulated between tumors and normal brain. Pathway analysis revealed significant enrichment (p < 0.05) for TGFβ, Ephrin receptor and Wnt/β-catenin signaling in the *migration signature *while the *stationary signature *presented with significant over-representation of amino-acid metabolism genes (p < 0.05) (Figure 2D).

### Migration signature predicts migration velocity in vitro

**Figure 3:**
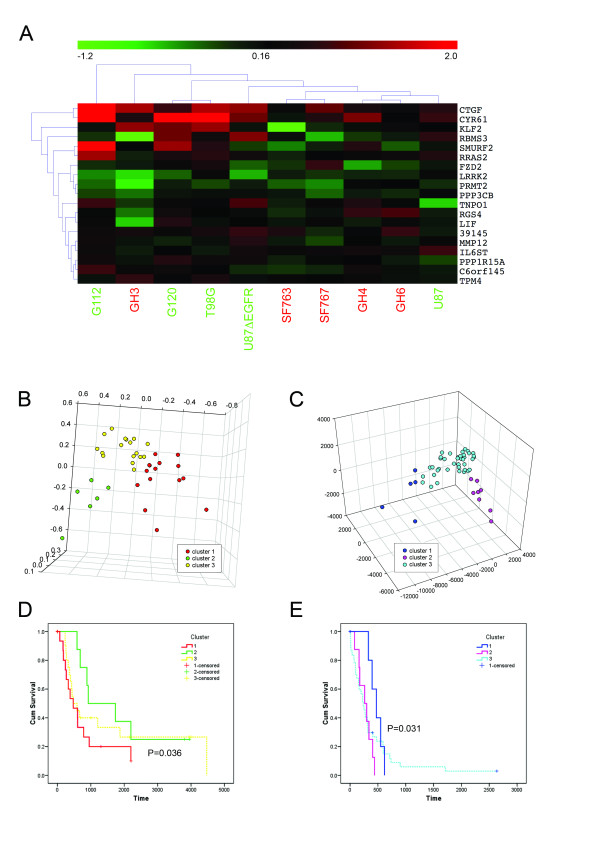
*Migration rate signature *derived from SAM analysis of union of *migratory *and *stationary signatures*; fast migrating cell lines colored in green, slow migrating cell lines colored in red (**A**). K-means (k = 3) identified three populations in malignant astrocytoma samples stratified by age that were visualized by multidimensional scaling (MDS); young (< median age) (**B**) and old patients (> median age) (**C**). Kaplan-Meier survival analysis for clusters derived from k-means stratified by age; young (**D**), old (**E**) show significant differences in overall survival.

Significance analysis of microarrays (SAM) was carried out on the union of *migratory *and *stationary signature *to select features that discriminate between fast (G112, G120, T98G, U87, U87ΔEGFR) and slow migrating glioma cells (GH3, GH4, GH6, SF763, SF767) (Figure 3A). A *migration rate signature *of 22 genes was found (delta = 0.188, FDR = 50%) and classifiers developed using support vector machines correctly predicted migration rate in 9 out of 10 glioma cultures in this study (permutation test P = 0.03). Cox hazard analysis identified CTGF as the gene with the highest regression coefficient with migration rate (0.17, P = 0.033).

### Migration rate signature predicts survival of glioma patients

Mapping the 22 genes (Agilent platform) from the migration rate signature to the clinical data set (Affymetrix) found that 19 genes were represented in the clinical data. Clinical samples were binarized according to patient by age (young < median age < old) and K-mean (k = 3) identified three distinct populations in each age group. Multidimensional scaling was used to visualize the clusters with 3D scatterplots (Figure 3B, C). In young patients (< 50 years) significant survival difference between clusters 2 and 3 were detected with a median survival of 924 days and 473 days, respectively (P = 0.036)(Figure 3D). In patients > 50 years of age, a significant difference was shown between cluster 3, median survival of 473 days, and cluster 2, median survival of 270 days (P = 0.031)(Figure 3E).

### Candidate Validation

#### Immunohistochemistry

**Figure 4:**
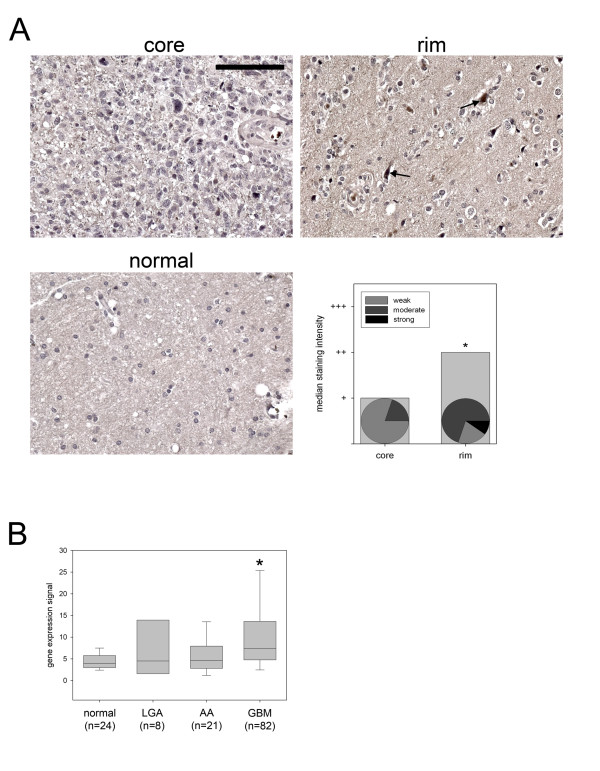
Immunohistochemistry of matched glioma core and rim samples as well as normal brain control from a glioma invasion specific tissue microarray (TMA). Staining for CTGF exhibits strong signal in a majority of invasive cells (rim, arrows) compared to stationary cells from tumor core. Normal brain control shows only weak staining; size standard = 200 μm. Median staining intensity for CTGF assessed separately in core and rim cells is represented as bar chart; pie charts represent staining intensity for respective portion of glioma cells separately in core and rim (*, p < 0.05) (**A**). Boxplot representing levels of CTGF in glioma expression data set (*, p < 0.05)(**B**).

A glioma invasion TMA was employed to investigate *in vivo *protein expression of CTGF. IHC staining intensity in seventeen GBM samples represented by matched tumor core and invasive rim from the same sample were evaluated by a pathologist (S.N.). Invasive glioma cells were found to exhibit significantly stronger median immuno-positivity for CTGF than stationary core cells (P = 0.025). Ten percent of invasive glioma cells showed strong staining for CTGF, 70% exhibited medium and 20% weak immuno-positivity while 80% of glioma cells in core samples exhibited weak staining and 20% exhibited medium staining. Normal brain astrocytes and neurons showed no staining for CTGF (Figure 4A).

Whole genome expression profiling of a series of human brain tumor specimens revealed CTGF expression to be significantly elevated in GBM (n = 82) compared to normal brain specimens (n = 24) (p < 0.004) (Figure 4B).

#### Functional validation

**Figure 5:**
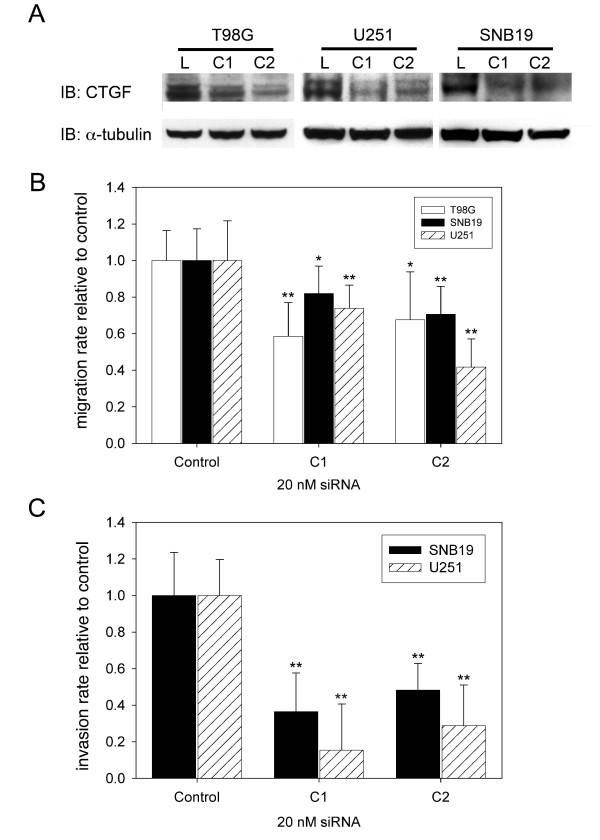
T98G, U251 and SNB19 glioma cells transiently transfected with two independent siRNAs against human CTGF (C1, C2) or luciferase show decreased protein levels in siRNA treated cells (A). CTGF knock-down results in decreased cell migration. Migration rate is expressed relative to luciferase (Control) treated samples (**A**). Cell invasion in organotypic rat brain slices; SNB19 and U251 glioma cells stably expressing GFP were transfected with siRNA directed against luciferase (control) or CTGF (C1, C2); z-axis invasion was assessed by confocal microscopy. Results are normalized to control-treated cells and all experiments were performed at least three times (**C**); Bars, SE; (*, p < 0.05; **, p < 0.001).

To assess biological significance of migration candidates for glioma migration siRNA knock-down was performed by transient transfection of T98G, SNB19 and U251 glioma cells with two independent siRNAs against CTGF (C1, C2). QRT-PCR revealed 90% reduction of CTGF mRNA levels as compared to control (data not shown) and immunoblotting confirmed significant reduction of protein levels compared to control in three glioma cell lines (Figure 5A). siRNAs against CTGF lead to significant reduction of *in vitro *glioma migration (inhibition of up to 41% in T98G, p < 0.001, up to 30% in SNB19, p < 0.01 and up to 58% in U251, p < 0.001) as compared to luciferase control (Figure 5B).

To evaluate biological effects of CTGF on glioma cell invasion through physiologically and anatomically relevant tissue, we determined whether RNA interference-mediated depletion of CTGF inhibits the invasion of GFP-expressing SNB19 and U251 human glioma cells into vital rat brain slices, a well-established organotypic model for glioma invasion. Cells treated with two independent siRNAs against CTGF or luciferase were implanted in 4 to 6 replicates on contralateral sides of the same rat brain slice and invasion was quantified by confocal microscopy. Depletion of CTGF in SNB19 causes significantly reduced cell invasion (C1: 37% ± 21, p < 0.001; C2: 48% ± 15, p < 0.001) compared with luciferase treatment (100% ± 24). Likewise, depletion of CTGF in U251 causes significant reduction in invasion (C1: 15% ± 25, p < 0.001; C2: 29% ± 22, p < 0.001) compared to cells treated with luciferase siRNA for control (100% ± 20) (Figure 5C).

## Discussion

In the present study we aimed at shedding light on the transcriptional mechanisms activated in glioma cells under migratory conditions and identifying gene candidates that can be translated into targets for anti-invasive therapies. A comprehensive panel of human glioma cell lines and primary cultures was employed for this study. To reflect already well-established genetic properties of gliomas, cell lines were selected according to their p53 status. In greater than 65% of secondary GBM (recurring) cases tumors present with p53 mutations [24]. Primary GBM exhibit overexpression and/or amplification of EGFR in about 60% of cases [25]. While these genetic aberrations are not strictly exclusive for primary or secondary GBMs, they are mutually exclusive to each other. We selected cell lines that express p53WT as reporters for primary glioblastomas (SF767, U87, U87ΔEGFR, G120) and ones with p53 mutations approximating secondary tumors (T98G, G112MS, SF763) [26]. To introduce heterogeneity of glial tumors that is frequently lost during long-term tissue culture three primary cultures (GH3, GH4, GH6) were included in this study.

While gene expression profiling has been widely used to identify subtypes of gliomas and to discern genes related to tumor progression and patient survival [27,28], only few studies have investigated the migratory or invasive phenotypes of tumor cells in-vitro [6,29]. We recently reported the invasive transcriptome of human gliomas by expression microarray and identified candidates driving the invasive phenotype *in vitro *and *in situ *[7]. Here, a novel discovery approach was taken to characterize the migratory phenotype of multiple glioma cell lines *in vitro *to allow for candidate discovery and functional validation. Whole human genome oligonucleotide microarray was employed to analyze gene expression profiles of migratory and stationary cells isolated from 7 established glioma cell lines and three primary cultures of human glioma biopsies. To ensure statistical rigor of this study, samples presenting migratory and stationary phenotype were collected in a randomized fashion as three independent biological replicates. These replicates provided sufficient statistical power for a 1.5-fold sensitivity between migratory and stationary cells. Quality control through technical replicates ensured that, as expected, technical variation in the array data was lower than biological variation between samples or cell lines.

As a first step of data analysis, genes that were not differentially expressed between phenotypes (i.e. stationary vs. migratory) were filtered through one-way ANOVA performed across all samples. We sought a pattern of individual genes responsible for differences between these phenotypes that may be involved in directing cell behavior. In a simplistic approach, we identified up-regulation and down-regulation patterns to find subsets of genes with correlated expression. PCA was employed to reduce high dimensional array data into components that represent the majority of variation within the data. All 20 PC scores were examined even though the patterns of interest (rim vs. core) were thought to represent only a small proportion of variation, which should materialize in higher components. Visual analysis of PCA scores yielded anticipated signatures in the higher PCs, specifically PC 9 and 10 representing a small fraction (3.4% and 2.9%, respectively) of the total variation. This observation indicates that the majority of variation within the expression changes in the dataset is a consequence of intrinsic differences between cell-lines as manifested by the hierarchical clustering of cell lines, in which core and rim samples of individual cell lines cluster together before biological (phenotypic) replicates of core and rim cluster together. A pattern describing down regulation in migratory cells compared to stationary cells was observed in PC9 and AK098354 was selected as a proband exhibiting strong correlation with this pattern. Similarly, CYR61 was chosen as a proband that significantly correlates with the pattern (PC10) for genes up-regulated in migratory cells. In a pattern-based approach implemented in GABRIEL, two gene signatures highly correlated (r > 0.8) with either proband, AK098354 or CYR61, were identified and termed *stationary *and *migratory signatures*. Based on low false discovery rates and concurrent high false negative rates it was concluded that the selected subsets contained genes that truly exhibited the desired up- or down-regulation pattern and merited further investigation.

The expression of genes in the stationary and migratory signatures was investigated in a more recently collected and larger *in vivo *gene expression study where stationary and invasive glioma cells were collected by laser-capture microdissection from 19 glioblastoma multiforme biopsy samples (unpublished data). We found that 51% (54 out of 105) of the genes in the stationary signature were down-regulated and 39% (21 out of 54) of genes in the migratory signature showed up-regulation in invasive tumor cells. CTGF was found to be up-regulated in invasive cells from 8 out of 19 tumor samples. Expression of the migratory signatures was further examined in these samples. A slightly lower number of stationary signature genes was found to be down-regulated (52 out of 105 – 49%), and 50% of migration signature genes were up-regulated (27 out of 54). This correlation between *in vitro *and *in vivo *motility gene expression profiles suggests a parallel between migration and invasion.

Literature mining was performed to identify genes whose functions were linked to cancer, migration and invasion; these were further evaluated in clinical datasets and in empiric testing for involvement in migration and invasion. Technical validation of 8 candidate genes by QRT-PCR confirmed directionality of array data (i.e. up- or down-regulation with migration). Incomplete concordance of RRAS, RhoA, PCAF and MADH1 regulation across all cell lines studied, was interpreted as biological variation of gene expression between cell lines reflecting the genetic diversity of the disease (Figure 2).

The biological significance of the *migratory *and *stationary signatures *is underscored by discovering that 75% of genes in the up-regulation signature were also found to be differentially regulated in expression datasets of human glial tumors compared to normal brain. In conjunction with overexpression of TGFβ, Ephrin receptor and Wnt/β-catenin signaling in migrating glioma cells we postulate that genes from the migratory signature act as drivers of invasion, a hallmark of astrocytic tumors. Also, these pathways have been linked to glioma migration in hypothesis driven experiments and therefore underscore the validity of our discovery approach (reviewed in [5,30-32]). Clinical relevance of the *stationary signature *lies in the significant enrichment for methionine metabolism. In PET studies of brain tumors, metabolism of C11-methonine is measured as a surrogate for protein synthesis [33] and is significantly enhanced in tumor tissue compared to normal brain [34,35]. Enhanced protein synthesis might therefore be a function of rapidly dividing tumor cells that are found predominantly in the stationary population [6]. Based on our recent work describing the phenotypic dichotomy between migratory and stationary glioma cells, dramatic differences in pathway activation as depicted by the lack of overlap in the pathway enrichment analysis between the two signatures are not unexpected [6,36]. Instead the expression profile of the migration assay is a faithful representation of the biology found in invasive tumors.

Interestingly, from the *migratory *and *stationary signatures *we could develop a classifier that predicts the migration rate of cell lines studied. It underscores that a gene signature might directly govern the invasive and metastatic potential of a tumor. While it has been demonstrated before, that modifications of the extracellular matrix and changes in the expression of single genes result in changes to migratory behavior [11,37], to our knowledge no comprehensive gene expression pattern that directs migration rate has been reported.

After stratification for age, the most important predictor for survival [38], the *migration rate signature *identified groups of patients that had significant differences in survival, underscoring the clinical significance of the *migration rate signature*. Hazard analysis revealed CTGF as the gene with the highest contribution to the migration rate. CTGF was initially identified as a mitogen, produced by vascular endothelial cells promoting proliferation and differentiation of chondrocytes [39]. CTGF has also been implicated in malignancy of chondrosarcomas [40], as well as in survival and grade of astrocytic tumors [41]. While CTGF has been reported to be a driver for invasion, this effect was at least in part attributed to effects on angiogenesis [42,43]. Our study for the first time attributes CTGF a more direct role in tumor cell migration and invasion as it was identified and validated in systems devoid of angiogenesis. The role of CTGF as an invasion related protein is further supported by the observation that it is significantly up-regulated in invasive gliomas in-situ: TMA of 24 invasive gliomas exhibited significant over-expression of CTGF in invasive glioma cells compared to stationary cells in core samples; normal brain tissue showed no staining for CTGF. In an expression data set derived from 111 human gliomas and 24 normal brain controls we found that CTGF is significantly up-regulated in GBM compared to control, a finding supporting our immunohistochemistry results, where normal brain tissue had no detectable staining for CTGF. Extending the findings made by Xie and coworkers [41] the observations mentioned above render CTGF a marker for invasive high-grade gliomas. While Xie et al. reported that CTGF is a predictor of patient survival we were not able to recapitulate this finding in three independent glioma expression data sets.

To test the functional role of CTGF in glioma migration, we performed knockdown studies with two independent siRNAs that yielded significant reduction of *in vitro *migration in three human glioma cell lines. This finding is further supported by the observation that CTGF knockdown results in decreased invasion of two human glioma cell lines in *ex vivo *organotypic rat brain slice assay, a system closely resembling extracellular matrix environment present in the brain. The observation that CTGF knockdown does not completely inhibit migration and invasion in our model systems underscores the finding in this manuscript that a network of genes, the migratory signature rather than a single gene, is the driver of the motile phenotype [13,44]. An alternate explanation for this observation might lay in the fact that long term cell lines do not assemble the highly invasive phenotype in vivo that is observed clinically [45] and therefore might not be as responsive to inhibition of key signaling molecules.

## Conclusion

The novel discovery approach presented here identified signatures of *migratory *and *stationary *glioma cells as well as a *migration rate signature*. The most prominent contributor to the migratory phenotype was validated clinically and functionally by siRNA. Taken together these findings provide strong rationale for anti-invasive therapies targeting CTGF, as this molecule was found to be significantly over-expressed in invasive glioma cells where knock-down studies confirmed biological activity.

## Authors' contributions

TD and JLR conceived, designed, carried out the experiments, analyzed the data and drafted the manuscript. DBH, LBR and MN designed and carried out experiments, analyzed data and drafted the manuscript. CB, SN, EMA, ANH and AJ carried out experiments and analyzed data. WS carried out migration assays. RL, KHP and CJL discussed data analysis, SNC discussed data analysis and the manuscript. MEB conceived and designed the study and drafted the manuscript. All authors read and approved the final manuscript.

## Supplementary Material

Additional file 1 Expression signatures of stationary and migratory glioma cells. Provided are details for stationary, migratory and migration rate signature derived from 10 human glioma lines. Click here for file
